# Vibrio vulnificus Causing Severe Multiorgan Failure in a Patient With Underlying Cirrhosis

**DOI:** 10.7759/cureus.84728

**Published:** 2025-05-24

**Authors:** Brandon Y Boeur, Hussein Haidari, Kristen Buchanan, Sagar Kumar, Philip Almalouf

**Affiliations:** 1 Internal Medicine, University of South Alabama College of Medicine, Mobile, USA; 2 Pulmonary and Critical Care, University of South Alabama University Hospital, Mobile, USA

**Keywords:** cirrhosis, dic, sepsis, splenic embolization, vibrio vulnificus

## Abstract

*Vibrio vulnificus* is a marine-associated gram-negative bacillus with the potential to cause severe sepsis. *V. vulnificus* is classically associated with interactions with shellfish, be it due to inoculation of the bacteria via shell laceration or ingestion of raw shellfish. Patients with underlying liver disease are prone to more severe outcomes associated with *V. vulnificus* infections. In this case, a male patient in his late 30s with chronic alcohol use disorder presented with acute encephalopathy and hematemesis. History revealed ingestion of raw oysters days prior to presentation. After admission, he developed severe multiorgan failure as well as severe persistent thrombocytopenia. He was started on antibiotic therapy with intravenous ceftazidime and oral doxycycline. He underwent partial splenic embolization with improvement in thrombocytopenia and eventual medical stabilization. It is important for clinicians to recognize classic risk factors for *V. vulnificus* infection to guide timely diagnosis and management.

## Introduction

The marine Gram-negative bacillus *Vibrio vulnificus* can present as primary septicemia or primary wound infections. Over 1000 cases occurred in the United States in 2013. Shellfish, particularly clams and oysters, are the primary source of *V. vulnificus*. After consumption of raw shellfish, patients may develop primary septicemia from gastrointestinal (GI) tract manifestations of the pathogen [1]. Acute fever, chills, hemorrhagic bullous lesions, and septic shock are the main clinical signs of a *V. vulnificus* infection. Death can occur rapidly in severe cases of *V. vulnificus* infection. According to the Food and Drug Administration (FDA), the death rate from *V. vulnificus* infections can reach 50% even in the United States [2]. Early diagnosis and treatment have a major impact on the prognosis of patients. *V. vulnificus* exposure or ingestion can cause disease as soon as four hours later, and nearly all patients who do not receive appropriate treatment within three days die [3]. The majority of *V. vulnificus* infection cases happen between March and November, particularly during the summer when the sea surface water temperature is between 23°C and 29°C or 37°C, which is ideal for growth [4]. Climate change-induced increases in sea surface temperatures are causing the endemic region of *V. vulnificus* to grow yearly [5]. Clinicians practicing in maritime areas should keep *V. vulnificus* infection in their differential.

## Case presentation

A 38-year-old Hispanic male patient with a medical history of cirrhosis due to alcohol presented to the emergency department with fever and hematemesis starting two days ago. Due to the patient's acute encephalopathy, collateral history was obtained by family. The family reported the patient had consumed raw oysters approximately the day before the patient began to have symptoms. The family also reported patient had a heavy drinking history with daily consumption of 14 beers. On arrival, the patient had vital signs concerning for sepsis as presented in Table 1.

**Table 1:** Initial Laboratory Results on Admission

	Patient values	Normal range
Ammonia	126 µg/dL	15-45 µg/dL
Platelets	15 ×10³/µL	150-450 ×10³/µL
White blood count	21 ×10³/µL	4.5-10.5 ×10³/µL
Total bilirubin	10 mg/dL	0.2-1.2 mg/dL
Lactate	8.4 mmol/L	0.5-2.2 mmol/L
International normalized ratio	3.36	0.8-1.2

**Figure 1:**
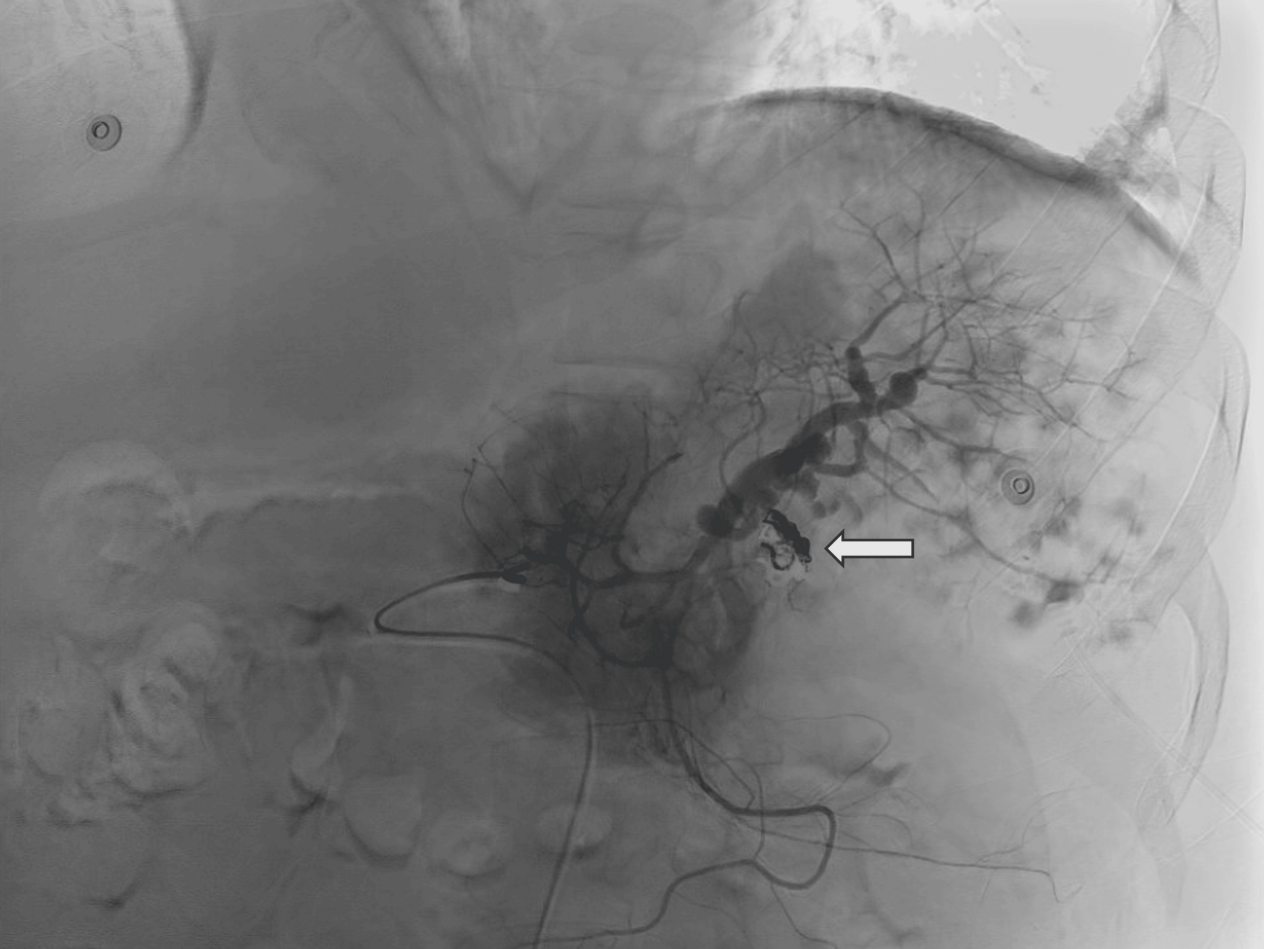
Fluoroscopic angiogram showing coil embolization of splenic artery Coil embolization of the splenic artery as noted by the white arrow. Intravenous contrast was used to visualize the vasculature.

At the initial clinician evaluation, the patient was acutely altered, with loss of orientation to person, place, and time. His vital signs were concerning for sepsis with hypothermia and tachycardia. His lab work was significant for elevated ammonia, thrombocytopenia, leukocytosis, elevated total bilirubin, and elevated lactate (Table 1). The patient was dyspneic as well as hypoxic and required supplemental oxygenation with 4L nasal cannula. Physical exam revealed icteric sclera, mildly distended abdomen, and diminished breath sounds from decreased respiratory effort. Blood cultures obtained at the time of admission cultured *V. vulnificus*. The patient was started on intravenous ceftazidime and oral doxycycline. Due to his thrombocytopenia, he was transfused with multiple units of platelets with mild improvement of platelets. However, despite continued supportive transfusions, the patient's thrombocytopenia persisted. Further investigations revealed elevated immature platelet fraction. Due to these findings, there was concern for splenic platelet consumption and interventional radiology was consulted for partial splenic embolization. The patient had partial splenic embolization four days after admission, seen in Figure 1. The patient had subsequent sustained increase in platelet count post-procedure and no longer required continued platelet transfusions, shown in Table 2. In addition, the patient began to improve clinically with resolving encephalopathy and was eventually discharged after one week. Three days after discharge, the patient returned to the hospital for signs of decompensated cirrhosis with labs consistent with multiorgan failure and severe coagulopathy with extremely elevated international normalized ratio (INR) with spontaneous bleeding in his mucosal cavity. He was started on continuous infusions of norepinephrine and vasopressin for persistent refractory hypotension. However, the patient later expired from multiorgan failure.

**Table 2:** Post partial splenectomy platelets

	Patient values	Normal range
Platelets	60 ×10³/µL	150-450 ×10³/µL

## Discussion

This case study highlights the severe consequences of *V. vulnificus* infection in a patient with advanced alcoholic cirrhosis. The patient’s chronic liver disease, ongoing alcohol consumption, and high-risk seafood consumption increased the lethal risk associated with raw oyster consumption in cirrhotic patients. Patients can contract *V. vulnificus* by eating contaminated seafood or getting it through open wounds [6]. Since iron is necessary for bacterial growth, people with liver dysfunction are more prone to infection because of their weakened immune systems and increased iron [7,8].

The patient's fever, encephalopathy, and gastrointestinal symptoms are all classic signs of *V. vulnificus* infection [3]. Rapid onset and disease burden are characteristics of this bacterial illness. The immune system dysfunction brought on by cirrhosis makes these patients more susceptible to infections due to mechanisms such as diminished opsonization and altered neutrophil activity [9,10]. The mortality rates for *V. vulnificus* sepsis in cirrhotic individuals are startlingly high, often exceeding 50%. The case described is consistent with the fact that even small bacterial loads can cause serious complications such as coagulopathy and multiorgan failure [11,12].

Splenic sequestration, bone marrow suppression, and changes in the hemostatic environment are some of the causes of thrombocytopenia in patients with cirrhosis [13]. In this case, a partial splenic embolization required an interventional radiology consultation due to significant refractory thrombocytopenia. This procedure effectively reduces splenic platelet consumption and increases circulating platelet counts [14]. After embolization, the patient's mental state improved and their platelet count momentarily stabilized, suggesting that the infection was initially under control and that their liver function had temporarily normalized. Additionally, improving outcomes in *V. vulnificus* infections requires the rapid initiation of medicines, particularly doxycycline, fluoroquinolones, and third-generation cephalosporins. The need for early detection and vigorous treatment in cirrhotic patients exposed to coastal environments is highlighted by the fact that delays in appropriate treatment might result in rapid deterioration [9,11,15].

The patient's continued decline following discharge, however, emphasizes the complex interaction between cirrhosis and sepsis, where multiorgan failure is still a serious concern and hepatic recovery is difficult [16]. A recurrence of multiorgan failure, significant coagulopathy, and spontaneous bleeding suggests systemic decompensation and irreversible liver disease development. A poor prognostic indication in cirrhotic patients is frequently coagulopathy, which is made worse by an elevated INR and the inability to stabilize in spite of vasopressors [17]. Laboratory investigations such as prothrombin time (PT), INR, partial thromboplastin time (PTT), and platelet count are important to obtain during evaluation.

Numerous pathophysiological pathways impact the interaction between infection and cirrhosis. Cirrhosis-associated immune dysfunction (CAID), which includes both increased inflammation and immunological suppression, is one important contributing factor. Immune responses are hampered by liver dysfunction in cirrhosis, which upsets the balance of cytokines and proteins that modulate the immune system. The immune response against infections is further weakened by decreasing complement protein levels and Kupffer cell activity [10]. The dysfunction of the immune system and decrease in circulating immune cells, particularly neutrophils, render individuals susceptible to severe infections, notably from Gram-negative bacteria like *V. vulnificus*. This is reflected in the impaired initial immune responses and prolonged pathogen clearance in those with cirrhosis [12,18].

Systemic inflammation, marked by activated circulating immune cells and increased serum concentrations of pro-inflammatory cytokines, results from the ongoing activation of immune cells stimulated by damage-associated molecular patterns (DAMPs) released from dying liver cells. As cirrhosis progresses, these DAMPs are also released from the compromised gastrointestinal tract, which further exacerbates the inflammatory response [10]. Portal hypertension resulting from cirrhosis leads to bacterial translocation, where intestinal bacteria and endotoxins enter the bloodstream, often initiating a systemic inflammatory reaction. In cases of advanced liver disease, this translocation results in low-grade endotoxemia, which can quickly escalate to sepsis when there is exposure to *V. vulnificus* [19,20].

Additionally, the impaired hemostasis seen in cirrhosis, marked by a lack of clotting factors, complicates the management of patients during sepsis by increasing the likelihood of bleeding. Metabolic issues, such as elevated ammonia levels and cognitive decline, can obscure infection symptoms, prolonging treatment duration and worsening the prognosis [20].

This interplay of immune dysfunction, impaired hemostasis, and systemic inflammation makes patients with cirrhosis especially vulnerable to severe consequences from infections caused by *V. vulnificus* [11].

## Conclusions

This case underscores the urgent necessity for preventive measures and patient awareness, especially among those with cirrhosis, about the dangers of consuming raw oysters. Healthcare providers should stress the necessity of steering clear of raw or inadequately cooked shellfish for individuals with liver disease, as this dietary decision can result in severe infections. The swift progression and high mortality associated with *V. vulnificus* sepsis should remind us of the importance of early diagnosis and treatment of infections caused by *V. vulnificus*. Clinicians should consider *V. vulnificus* in patients from coastal areas presenting with sepsis. Empiric agents which include a third-generation cephalosporin and doxycycline can potentially help reduce further escalation of infection. In addition, healthcare providers should take time to appropriately counsel patients with underlying hepatic dysfunction regarding the effects of continued alcohol consumption. Hepatic dysfunction can lead to impairment in the immune system, as well as other comorbid conditions, which could potentially delay the diagnosis of an underlying severe infection. Hepatic encephalopathy is a well-known complication of cirrhosis, which has similar presentation to patients presenting with severe infection.
